# Piperazine ferulate inhibits diabetic nephropathy by suppressing AGE/RAGE-mediated inflammatory signaling in rats and podocytes

**DOI:** 10.3389/fphar.2024.1394369

**Published:** 2024-08-01

**Authors:** Xiu-Meng Zhang, Xin-Ran Min, Hong-Xiao Xie, Yan-Ning Jiang, Yi-Xin Rui, Bo Li, Nan Zeng, Rong Liu

**Affiliations:** ^1^ State Key Laboratory of Southwestern Chinese Medicine Resources, School of Pharmacy/School of Modern Chinese Medicine industry, Chengdu University of Traditional Chinese Medicine, Chengdu, China; ^2^ School of Pharmacy, Chengdu University of Traditional Chinese Medicine, Chengdu, China; ^3^ Chengdu Hanpharm Pharmaceutical Co., Ltd., Pengzhou, China

**Keywords:** AGE-RAGE, diabetic nephropathy, inflammations, piperazine ferulate, podocyte

## Abstract

**Objective:** Diabetic nephropathy (DN) is a serious complication that may occur during the later stages of diabetes, and can be further exacerbated by podocyte damage. Piperazine ferulate (PF) has well-defined nephroprotective effects and is used clinically in the treatment of chronic nephritis and other kidney diseases. However, the renoprotective effects and mechanisms of PF on DN are not clear. This study aims to investigate the protective effect of PF on DN and its mechanism of action, to inform the clinical application of PF in DN treatment.

**Methods:** Network pharmacology was performed to predict the mechanism of action of PF in DN. Male Sprague Dawley rats were intraperitoneally injected with STZ (60 mg/kg) to establish a DN model, and then assessed for renal injury after 12 weeks of administration. *In vitro*, rat podocytes were treated with 25 mmol/L glucose and cultured for 24 h, followed by an assessment of cell injury.

**Results:** Our results showed that PF significantly improved renal function, reduced renal pathological changes, decreased inflammatory response, and alleviated podocyte damage in DN rats. PF also attenuated glucose-induced podocyte injury *in vitro*. Regarding molecular mechanisms, our study demonstrated that PF downregulated the expression of genes and proteins related to AGE-RAGE-mediated inflammatory signaling.

**Conclusion:** In summary, PF exerts its renoprotective effects by decreasing inflammation and protecting against podocyte injury through the inhibition of the AGE/RAGE/NF-κB/NLRP3 pathway. Overall, these data support the clinical potential of PF as a renoprotective agent in DN.

## 1 Introduction

Diabetic nephropathy (DN) is a serious complication that commonly occurs in the later stages of diabetes, and is a major cause of end-stage renal disease (Papadopoulou-Marketou et al., 2017; Thipsawat, 2021). DN has a high prevalence and is estimated to affect more than 50% of patients with T1DM(Wang et al., 2022). The development of DN leads to irreversible nephrin damage resulting in proteinuria, elevated blood pressure, and a sustained decrease in glomerular filtration rate (GFR)(Tonneijck et al., 2017; Ming, 2023). The high morbidity and mortality of DN present a significant public health challenge, leading to a poor quality of life for patients and imposing enormous social and economic burdens worldwide (Zhang et al., 2020). Currently, DN treatment primarily focuses on controlling diabetes and hypertension, as well as using ACEIs and ARBs, but the residual risk of DN remains significant. Novel drugs targeting glomerular hyperfiltration, inflammation, and other mechanisms have been a major focus for the development of new therapies (Alicic et al., 2017).

Chronic hyperglycemia leads to long-term damage, dysfunction, and failure of the kidneys, triggering necroinflammation and renal infarction, thereby exacerbating the loss of glomerular function (Karunasagara et al., 2020; Yang et al., 2023). Studies have shown that persistent hyperglycemia leads to lesions of the renal podocytes, which are typically characterized by cellular hypertrophy, foot process effacement, and podocyte depletion (Nakamichi et al., 2021). Podocytes are cells that cover the outside of the glomerular basement membrane (GBM) (Andrikopoulos et al., 2008), and play an important role in glomerular filtration; therefore, their damage leads to serious consequences. For example, proteinuria, a typical feature of renal disease, occurs even at the early stages of podocyte injury (Nagata, 2016). Podocyte damage is intricately linked to inflammation, and It has been shown that inhibition of NOD-like receptor protein 3 (NLRP3) inflammasome activation decreases renal inflammation and fibrosis in DN while attenuating podocyte damage (Wu et al., 2021).

Advanced glycation end products (AGEs) can trigger the activation of multiple signaling pathways via their action on a series of receptors, ultimately resulting in cellular impairment. AGEs accumulates in large quantities during the development of diabetes mellitus (DM), and the reduction of AGE production is beneficial to renal protection (Li et al., 2023). The receptor for AGEs (RAGE) is a classical AGE receptor, and AGE-RAGE binding can affect the initiation and activation of downstream signaling, including pathways relating to oxidative stress, inflammation, aging, and cancer (Han et al., 2023). The role of inflammation in the development of DN is a complex process involving multiple cytokines, chemokines, and inflammatory mediators, which exacerbate renal tissue damage by activating cell signaling pathways (Wada and Makino, 2013). Studies have shown that activation of the nuclear factor-κB (NF-κB) pathway is mediated by increased RAGE expression in the development of DN (Navarro-González et al., 2011; Rungratanawanich et al., 2021).

NF-κB is an important transcription factor in eukaryotes that regulates a wide range of cellular processes, including inflammation, the immune response, apoptosis, and growth and development (Ahmed et al., 2016). Activation of NF-κB leads to the downstream activation of the NLRP3 inflammasome, a multiprotein signaling complex integral to the chronic inflammatory response. NLRP3 activation in turn results in the maturation and release of the inflammatory cytokines interleukin 1 beta (IL-1β) and interleukin 18 (IL-18) (Williams et al., 2022). Thus, AGEs, RAGE, NF-κB, and the NLRP3 inflammasome are key factors regulating the inflammatory molecules associated with the pathogenesis of DN.

Piperazine ferulate (PF), known as 3-methoxy-4-hydroxy cinnamic acid piperazine, belongs to the non-peptide endothelin receptor antagonists. It is a chemical drug synthesized from ferulic acid and piperazine hexahydrate, based on the structure and action characteristics of ferulic acid and rhizoma chuanxiongxizine, the main components of the traditional Chinese medicine chuanxiong (Li et al., 2020). Currently, PF is used clinically in ttreating various chronic kidney diseases, such as chronic nephritis and nephrotic syndrome. PF is also commonly co-administered with ACEIs or ARBs, such as captopril and irbesartan, for the treatment of DN (DENG et al., 2018; Gu, 2020; LIN Jing et al., 2020). However, the efficacy and mechanism of action of PF alone in the treatment of diabetic nephropathy have not been reported. Our past studies have shown that PF attenuates acute kidney injury by activating NF-κB and the NLRP3 inflammasome (Li, 2022). Meanwhile, it has been reported that PF protects rats from cardiac ischemia-reperfusion injury by inhibiting the activation and cleavage of the NLRP3 inflammasome (Lei et al., 2022). Currently, drugs for treating DN, such as RAAS-targeting-drugs, have some therapeutic benefit but do not significantly prevent disease progression (Fu et al., 2019; Barrera-Chimal and Jaisser, 2020). In the present study, we sought to determine whether PF ameliorates DN by inhibiting the activation of AGE/RAGE-mediated inflammatory signaling and elucidates the relevant mechanisms. Our data supports the nephroprotective effects of PF and thus contributes to expanding the choices available for the pharmacological treatment of diabetic nephropathy to improve clinical outcomes.

## 2 Materials and methods

### 2.1 Materials and reagents

Piperazine ferulate (PF) was provided by Chengdu Hanpharm Pharmaceutical Co., Ltd (Sichuan, China). streptozotocin (STZ) was purchased from Sigma Aldrich (Shanghai) Trading Co., Ltd (Shanghai, China). Irbesartan dispersible tablets (referred to as IRB in this paper) were purchased from Sanofi Pharmaceutical Co., Ltd (Hangzhou, China) as a positive control drug.

### 2.2 Bioinformatics analysis

Existing databases were analyzed to identify key drug and disease targets. Since ferulic acid is the main component of piperazine ferulate, drug targets were collected using “ferulic acid” as the keyword in TCMSP (https://old.tcmsp-e.com/tcmsp.php), pharmmapper (http://www.lilab-ecust.cn/pharmmapper/) and other databases. Relevant literature was reviewed, and gene names were matched after merging and de-emphasizing the keywords. In Gene Cards (https://www.genecards.org/), OMIM (https://omim.org/), TTD (https://db.idrblab.net/ttd/) and other databases, “diabetic nephropathy” were used as the keyword to collect disease targets, which were then combined and de-emphasized to match the gene names. Venny 2.1 was used to obtain the intersection of the drug and disease targets.

PPI network construction and core target analysis were then performed. The hits identified at the intersection of drug and disease targets were uploaded into STRING 11.5, the species was set as “*Homo sapiens*,” and the predicted protein-protein interactions were obtained. The results were imported into Cytoscape 3.10.0 in TSV format for topological analysis of the network. Degree centrality (DC), betweenness centrality (BC), and closeness centrality (CC) were used to identify the core nodes for PPI network analysis. Core targets were obtained by screening with the condition that all DC, BC, and CC values were greater than or equal to the median.

The hits identified at the intersection of drug and disease targets were also imported into DAVID (https://david.ncifcrf.gov/) for GO and KEGG pathway enrichment analysis. The species was selected as “*H. sapiens*,” and the enrichment results were imported into Excel after analysis. The -log10 (*p*-value) columns were sorted in descending order, and the top 10 GO-enriched and KEGG-enriched entries were visualized, respectively.

### 2.3 Animals

Male Sprague-Dawley rats (weighing 220 ± 20 g, n = 30) were obtained from SiBeiFu (Beijing) Biotechnology Co., Ltd. (Beijing, China) (animal protocol: SCXK (Jing) 2019–0010. NO.110324220101763085). The rats were randomly divided into two groups: control (CON, n = 6), and STZ (STZ, 60 mg/kg, n = 24). The STZ group was injected intraperitoneally with STZ, while the rats in the CON group were treated with saline. Fasting blood glucose was measured from the tail vein 72 h after intraperitoneal injection of streptozotocin, and rats were considered diabetic when the fasting blood glucose (FBG) was ≥16.7 mmol/L (300 mg/dL). The 24 h proteinuria levels of rats was measured in the 8th week after induction of diabetes, and DN modeling was considered successful when 24 h UTP≥30 mg.

The rats with successfully induced DN were then randomized into 4 groups: the model group (MO, n = 6), IRB group (IRB, 16 mg/kg, n = 6), PF50 group (PF, 50 mg/kg, n = 6), and PF100 group (PF 100 mg/kg, n = 6). The concentration and dosage of PF and IRB were determined with reference to previous experiments (Lei et al., 2022; Li et al., 2022) and clinical dosage (Wei et al., 2016). Rats in the IRB, PF50, and PF100 groups were gavaged with PF or IRB for 12 weeks, while rats in the CON and MO groups were simultaneously given equal volumes of distilled water. Urine was collected from the rats every 2 weeks during this period. At the end of the treatment period, animals were anesthetized with sodium pentobarbital (40 mg/kg), and serum and kidney samples were collected for further analysis.

### 2.4 Assessments of biochemical parameters and routine blood test

The levels of serum creatinine (Scr) and blood urea nitrogen (BUN) (##141322005, #141122015, Shenzhen Mindray Biomedical Electronics Co., Ltd, China), and 24 h urine protein (24 h UP) (#C035-2-1, Nanjing Jiancheng Bioengineering Institute, China) were detected using specific kits following the manufacturer’s protocols.

### 2.5 Cell culture and treatment

Normal rat podocyte cells were purchased from Shanghai Hongshun Biotechnology Co., Ltd. and cultured in low-glucose DMEM supplemented with 10% FBS and 1% antibiotics (penicillin 100 IU/mL and streptomycin 100 mg/mL) in a humidified environment at 37°C with 5% CO_2_. In the preliminary study, rat podocytes were divided into five groups: the control group (CON), hypertonic group (D-Man, mannitol 25 mM) model group (MO, D-glucose 25 mM), IRB group (IRB, Irbesartan 20 μg/mL), and PF group (PF 12.5, 25, 50 μg/mL). After the cells reached 80% confluence, the D-mannitol group was stimulated with DMEM medium containing 25 mM mannitol for 24 h. Except for the blank and D-mannitol groups, all other groups were stimulated with medium containing 25 mM D-glucose for 24 h. After D-glucose stimulation, Irbesartan 16 μg/mL was added to the IRB group, and PF12.5, PF25, and PF50 μg/mL were added to the PF12.5, PF25 and PF50 groups, respectively, with the incubation continued for another 24 h.

### 2.6 Inhibitor

The NF-κB Inhibitor: BAY 11–7821 (#HY-13453, MEDCHEMEXPRESS LLC) is a selective and irreversible inhibitor of IκB-α phosphorylation. BAY 11–7821 (1.25 μM) or PF (50 μg/mL) was added to the culture medium after normal or high glucose treatment of podocytes for 24 h. Protein expression levels of p-NF-κB p65/NF-κB p65, p-IκBα, NLRP3 and Cleaved-IL-1β were detected by Western blotting.

### 2.7 ELISA analysis

The levels of TNF-α, IL-1β, IL-18, and IL-6 in serum (# FU01440J7162, # MM-0194R1, # MM-0047R1, # FU054ZDZ9154, Elabscience Biotechnology Co., Ltd, Jiangsu Meimian Ltd, Jiangsu Meimian Industrial Co., Ltd), as well as the content of cellular inflammatory factors such as TNF-α and IL-6 in cell supernatants were detected. Meanwhile, the levels of other components such as KIM-1 and CYS-C (# FU04D6D26986, # FU024T288262, Elabscience Biotechnology Co., Ltd) in the serum were also measured.

### 2.8 CCK8 assay

Renal podocyte cells treated with different concentrations of high glucose or mannitol were inoculated on 96-well plates with a density of 3.5 × 10^4^ cells/well. After incubation for 24 h, 100 μL CCK8 was added to the cells and incubated for 1 h. The OD value was detected by a microplate reader (Thermo Scientific, 3001) at 450 nm wavelength.

### 2.9 Western blot analysis

Total protein extracted from kidneys and cell lysates was quantified using a BCA protein assay kit (#P0010, Beyotime Biotechnology, Shanghai, China). Protein samples were separated on a preset 10% SDS-PAGE and then transferred to a nitrocellulose membrane in pre-cooled electrophoretic transfer buffer. Bands were cut according to the molecular weight of the target protein, and the target bands were washed with Tris buffer (TBS-T) containing 0.1% Tween-20 for 5 × 6 min. The membrane was then blocked with 5% BSA or skimmed milk for 90 min at room temperature. The primary antibody (AGEs:#bs-1158R,bioss; RAGE:# TA5309,abmart; nephrin: # TD7501S, abmart; NLRP3:#T55651, abmart; IKK alpha + beta; #T55660, abmart; Phospho-IKK alpha/beta (Ser176/Ser177); #TA3014, abmart; NF-κB p65: #T55034, abmart; Phosphor-NF-κB-p65: # AF 2006, Affinity; IκB alpha: T55026, abmart; Phospho-I kappaB- alpha (Ser32/Ser36): #TP56280, abmart; NPHS2:# TU390434, abmart; Total and Cleaved IL1b:#P50520-1R1, abmart) was incubated at 4°C overnight. On the next day, the strips were removed and the membrane was washed 5 times with TBST solution for 6 min/time; the secondary antibody was added and incubated for 90 min, and then the membrane was washed 5 times with TBST solution for 6 min/time. The membrane was incubated with developer solution and then exposed to a developer imager to develop the color, and the results of strip imaging were analyzed by Image Lab 6.0 software.

### 2.10 Histopathology

#### 2.10.1 HE&PAS staining

After euthanasia, the kidneys of each group of rats were dissected and rapidly fixed in 4% paraformaldehyde and embedded in paraffin. Thin slices of 3 μm were stained with hematoxylin and eosin (H&E) and photographed using a Pannoamic 250 digital section scanner (3DHISTECH). The histologic lesions of rat kidneys were observed under a microscope and scored according to the degree of kidney damage.

Similarly, paraffin-embedded kidney sections were also stained with PAS. Imaging was performed using a Pannoamic 250 digital section scanner, and the integrated optical density (IOD) and area of all captured images were measured using the Image Pro-Plus 6.0 image analysis system, and the percentage area of positive expression was calculated.

#### 2.10.2 Transmission electron microscopy

A small piece of kidney tissue was pre-fixed with 3% glutaraldehyde, rinsed with PBS, and post-fixed with 1% osmium tetroxide. After acetone gradient dehydration, ultrathin sections of approximately 60–90 nm were prepared and placed on a copper mesh grid. After the sections were stained, images of the copper mesh were acquired using a transmission electron microscope (JEM-1400FLASH).

#### 2.10.3 Immunofluorescence

For immunofluorescent tissue staining, paraffin-embedded kidney sections underwent antigen retrieval, and were then incubated in primary antibody (nephrin: # TD7501S, abmart; phospho-NF-κB-p65: # AF 2006, Affinity) overnight. The next day, sections were washed, the corresponding HRP-labeled secondary antibody was added, and the nuclei were counter-stained with DAPI. Coverslips were mounted and slides were sealed after adding fluorescence quencher, and images were collected.

To perform immunofluorescent staining of cultured cells, podocytes were fixed in 4% paraformaldehyde for 30 min, and then washed with PBS three times. The cells were permeabilized with 0.1% Triton X-100 for 15 min and blocked with 5% BSA for 1 h. The primary antibody (nephrin: # TD7501S, abmart) was incubated with samples overnight at 4°C. The next day, samples were incubated with the secondary antibody at 37°C for 1 h, then stained with DAPI for 5 min. Images were acquired using, an ultra-high-resolution imaging system.

### 2.11 Statistical analysis data

Data were analyzed using IBM SPSS 26.0 software. Results are expressed as mean ± standard deviation (SD) of at least four independent experiments. One-way analysis of variance (ANOVA) or non-parametric tests were used to assess whether differences between the experimental and control groups were statistically significant. Differences were considered statistically significant when the *p*-value was less than 0.05. All graphs were designed using GraphPad Prism software 8.0.

## 3 Results

### 3.1 Identification of potential targets of piperazine ferulate

**FIGURE 1:**
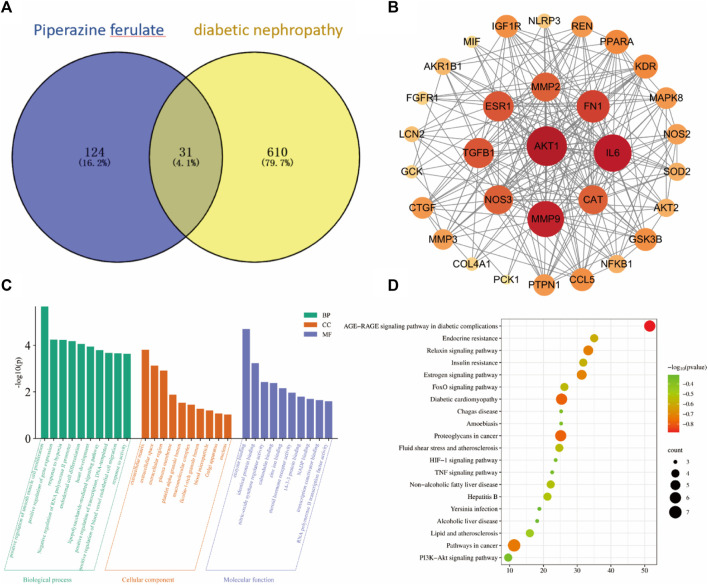
Bioinformatics analysis. venny diagram of the same target of drugs and diseases **(A)**. PPI Network Construction Core Target **(B)**. GO and KEGG Pathway Enrichment Analysis **(C, D)**.

Through database searches, we identified 124 targets for ferulic acid and 610 targets for diabetic nephropathy (DN), with 31 targets overlapping between the two. PPI network analysis identified a total of 31 nodes in the network. The nodes in the graph represent the target proteins, and the edges represent the interactions between the target proteins. The node size and color indicate the magnitude of the node’s importance; the larger nodes of darker color indicate greater importance. Notably, 10 core targets of ferulic acid in the treatment of DN were identified, such as AKT1, MMP9, IL-6, and TGF-β1 (Figures 1A,B).

The 10 key targets were imported into the DAVID database and subjected to GO function and KEGG pathway analysis. GO analysis identified 95 biological processes, which were mainly involved in the positive regulation of smooth muscle cell proliferation and the positive regulation of gene expression, among other processes. GO analysis also identified 10 cellular components, which were mostly related to the extracellular matrix and extracellular interstitial space, and 18 molecular functions, which were primarily involved in enzyme binding and homodimer formation (Figure 1C).

A total of 43 pathways were obtained from the KEGG enrichment analysis, and the signaling pathways were visualized using the Microbiology online mapping tool. The identified pathways included AGE-RAGE signaling and diabetic cardiomyopathy (Figure 1D).

### 3.2 PF improved renal function in rats with DN

**FIGURE 2:**
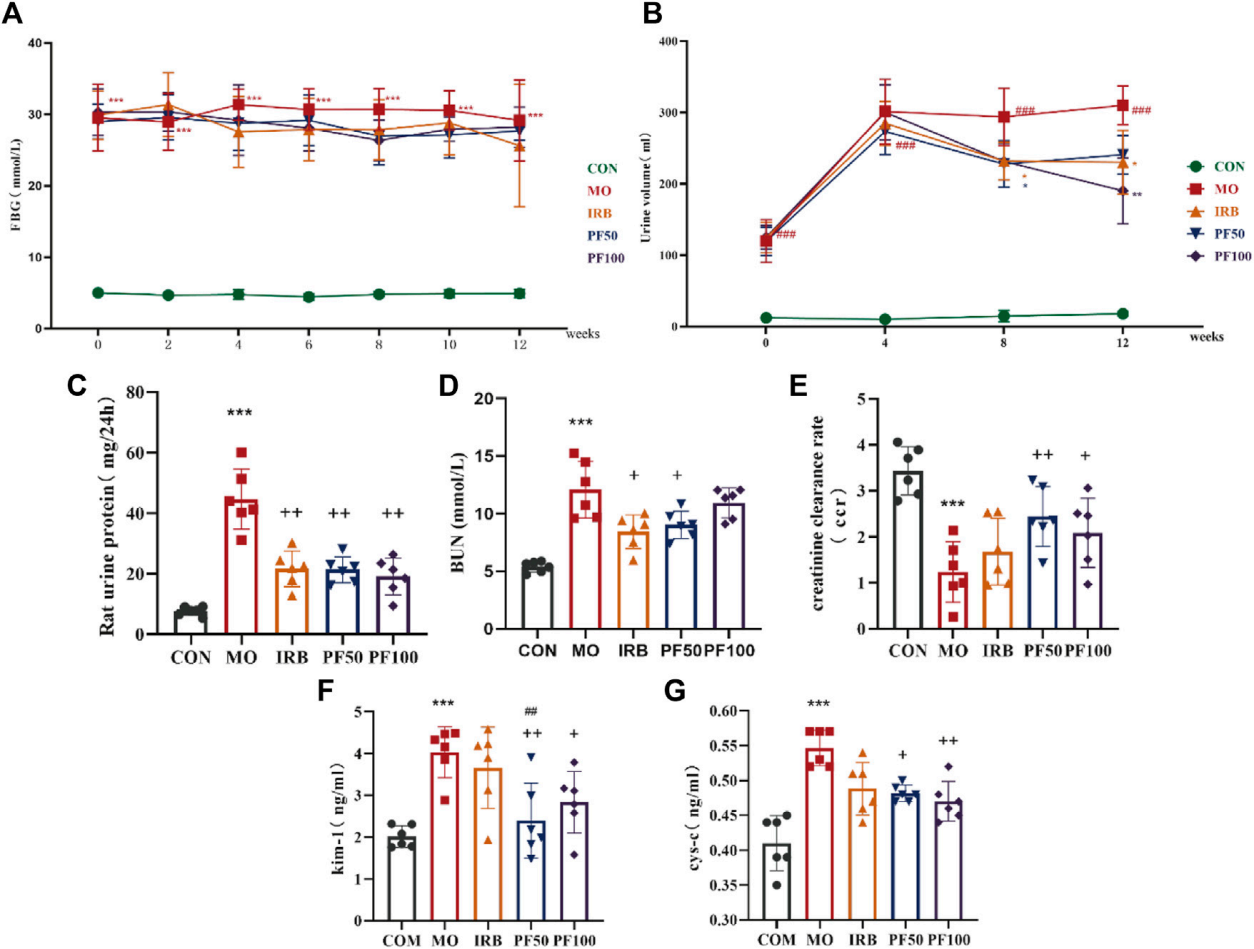
PF improves renal function in DN rats. Effect of PF on fasting blood glucose (FBG) **(A)**, Dynamic observation of PF on urine volume (UV) **(B)**, 24 h urinary protein (24 h-UP) **(C)**, blood urea nitrogen (BUN) **(E)**, creatinine clearance rate (CCR) **(D)**, Kidney Injury Molecule-1 (KIM-1) **(F)**, cystatin-c (cys-c) **(G)** in DN rats (n = 6). Data are expressed as ‾x±SD, ^*^ *p* < 0.05, ^**^ *p* < 0.01, ^***^ *p* < 0.001 relative to the control group; ^+^ *p*< 0.05, ^++^ *p*< 0.01, ^+++^ *p*< 0.001 compared to the model group; ##*p* < 0.01, compared to the IRB group.

Proteinuria is considered a hallmark of DN. Endogenous creatinine clearance (ccr) is used to estimate GFR and is commonly used as a proxy for the excretory function of the kidney (Wittmann et al., 2005; de Boer et al., 2007). Kidney injury molecule 1 (KIM-1) and Cystatin C (cys-c)are also markers of early kidney injury, and have been extensively studied in recent years. STZ-treated rats showed significantly increased FBG, urine volume and 24 h UTP, decreased CCR, and increased BUN, KIM-1, and cys-c. Therefore, sustained hyperglycemia successfully induced kidney injury in the rats. In contrast, in addition to having no effect on blood glucose, PF treatment improved all key renal parameters in DN rats, reflecting a beneficial therapeutic effect on the kidneys (Figure 2).

**FIGURE 3:**
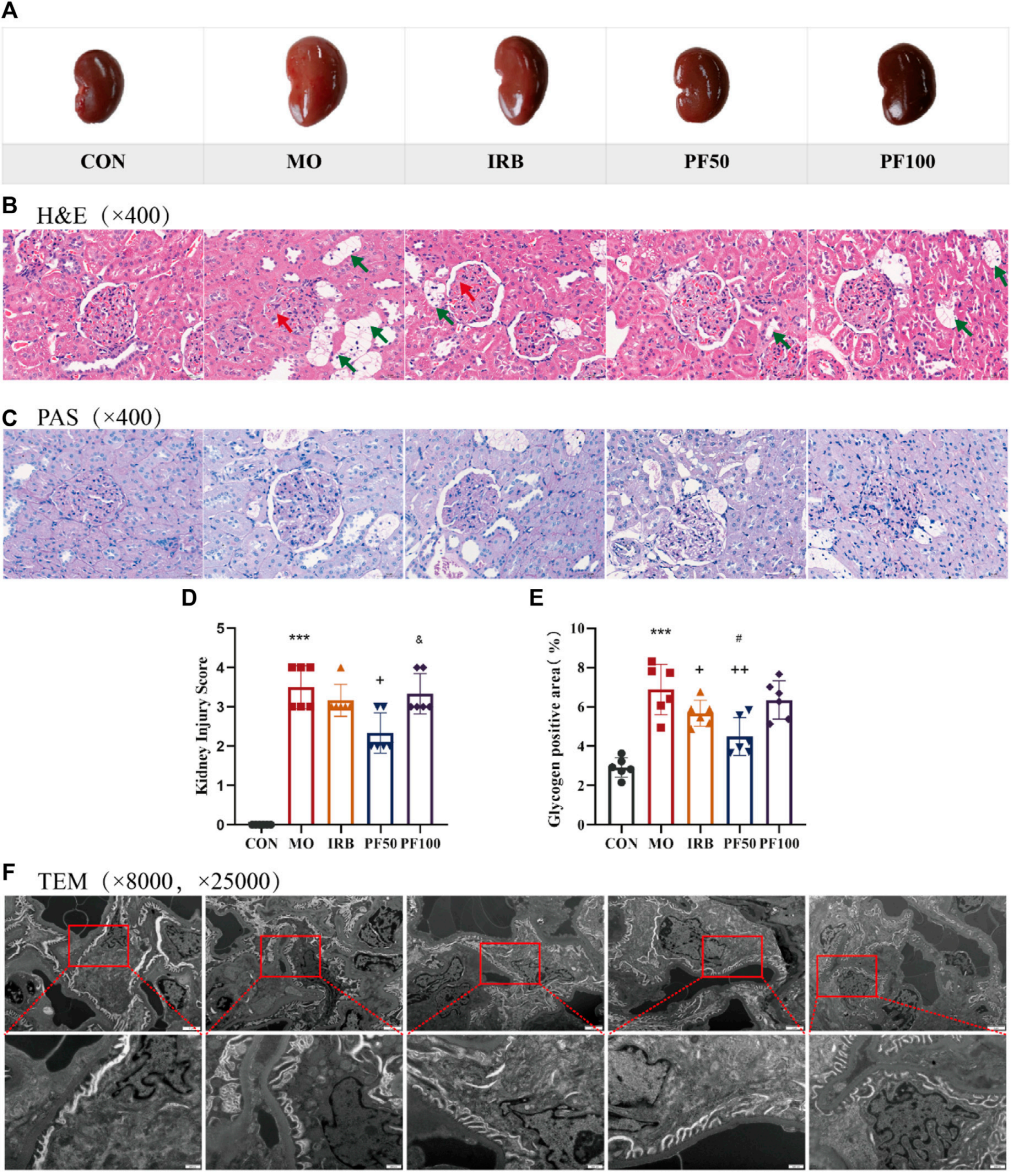
Histopathologic changes in the kidney. Representative macroscopic appearance of the kidney **(A)**. Kidneys were stained by H&E (400 ×) **(B)** and PAS (400 ×) **(C)**. Kidney damage score **(D)** and glycogen positive area **(E)**. Renal transmission electron microscopy (8000 ×, 25,000 ×) **(F)** (n = 4). Data are expressed as ‾x±SD, *p < 0.05, **p < 0.01, ***p < 0.001 relative to the control group; ^+^ *p*< 0.05, ^++^ *p*< 0.01, ^+++^ *p*< 0.001 compared to the model group; #*p* < 0.05, compared to the IRB group; ^&^ *p* < 0.05, compared to the PF 50 mg/kg group.

Histopathological changes were also investigated. The kidneys of rats in the model group appeared enlarged and lighter in color compared to healthy controls; this effect was reversed by PF treatment (Figure 3A). The results of H&E staining showed no obvious pathological changes in the control group. In contrast, the model group showed different degrees of basement membrane thickening in the glomeruli, with an increase in mesangial cells and deposition of mesangial matrix. A small number of renal tubular vacuoles were degenerated, and the nuclei of renal tubular epithelial cells were suspended in the center of the cells, with cytoplasmic lysis in a net-like pattern. However, PF treatment improved this phenomenon (Figures 3B, D). PAS staining was also performed to detect glycogen deposition in the tissue. Kidney damage scores and areas with PAS-positive staining were significantly reduced in PF-treated rats (Figures 3C, E).

To further explore the effect of hyperglycemia on glomeruli, we used transmission electron microscopy. In the control group, the foot processes of the glomerular podocytes were clearly observable, and the basement membrane was continuous and maintained a consistent thickness. In the model group, the glomerular filtration barrier had obvious lesions, some podocytes had undergone apoptosis, most of the mitochondria were mildly swollen, the morphology of the foot processes was abnormal, and there was observable foot process effacement in some areas. Furthermore, the basement membrane was thickened compared with that in the CON group, which was markedly improved by the administration of PF (Figure 3F).

### 3.3 PF attenuates glomerular podocyte injury in DN rats

**FIGURE 4:**
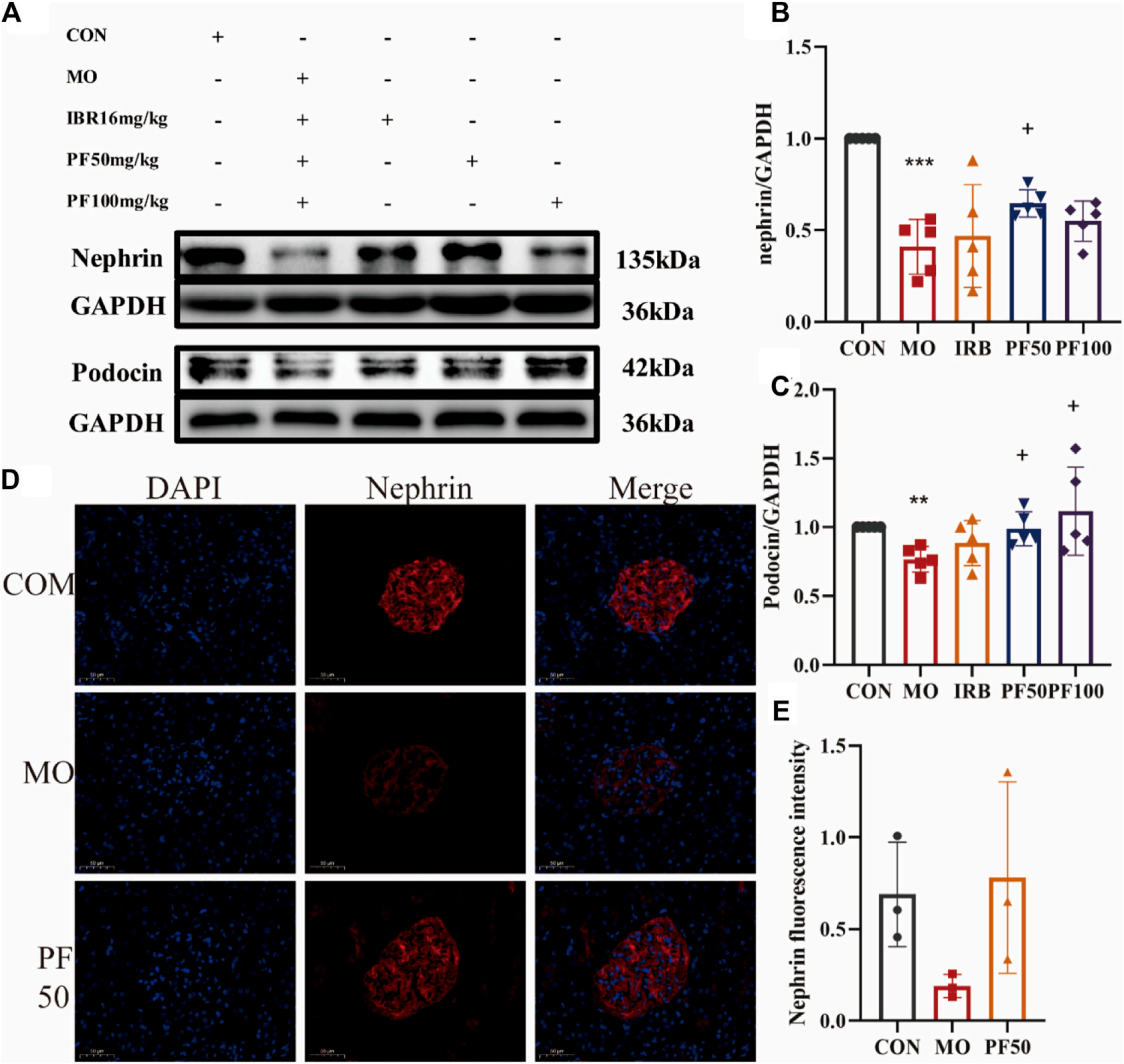
Effect of high sugar on glomerular podocytes in DN rats. The expression levels of nephrin and podocin proteins were determined by Western blotting **(A–C)** (n = 4). Immunofluorescence was used to detect nephrin protein expression (Calculate the average fluorescence intensity using ImageJ) **(D, E)** (n = 3). Data are expressed as ‾x±SD, *p < 0.05, **p < 0.01, ***p < 0.001 relative to the control group; ^+^ *p*< 0.05, ^++^ *p*< 0.01, ^+++^ *p*< 0.001 compared to the model group.

Nephrin is necessary for podocyte maturation and the formation of the slit diaphragm junction complex during kidney development, and podocin plays an important role in regulating tight junctions between adjacent podocytes. Because the expression of nephrin and podocin is downregulated in acquired glomerular diseases, the low levels of nephrin and podocin are considered pathological features of glomerular injury (Li et al., 2015; 2023). To investigate whether hyperglycemia can damage podocytes and tast the protective effect of PF on podocytes, we measured the levels of podocyte marker proteins nephrin and podocin in rat kidneys by Western blotting. The results showed that nephrin and podocin protein levels were reduced in the kidneys of rats in the model group, an effect that was reversed by PF. Similarly, immunofluorescent staining for nephrin expression in rat glomeruli yielded the same results as western blotting (Figure 4).

### 3.4 PF attenuates the inflammatory response in DN rats

**FIGURE 5:**
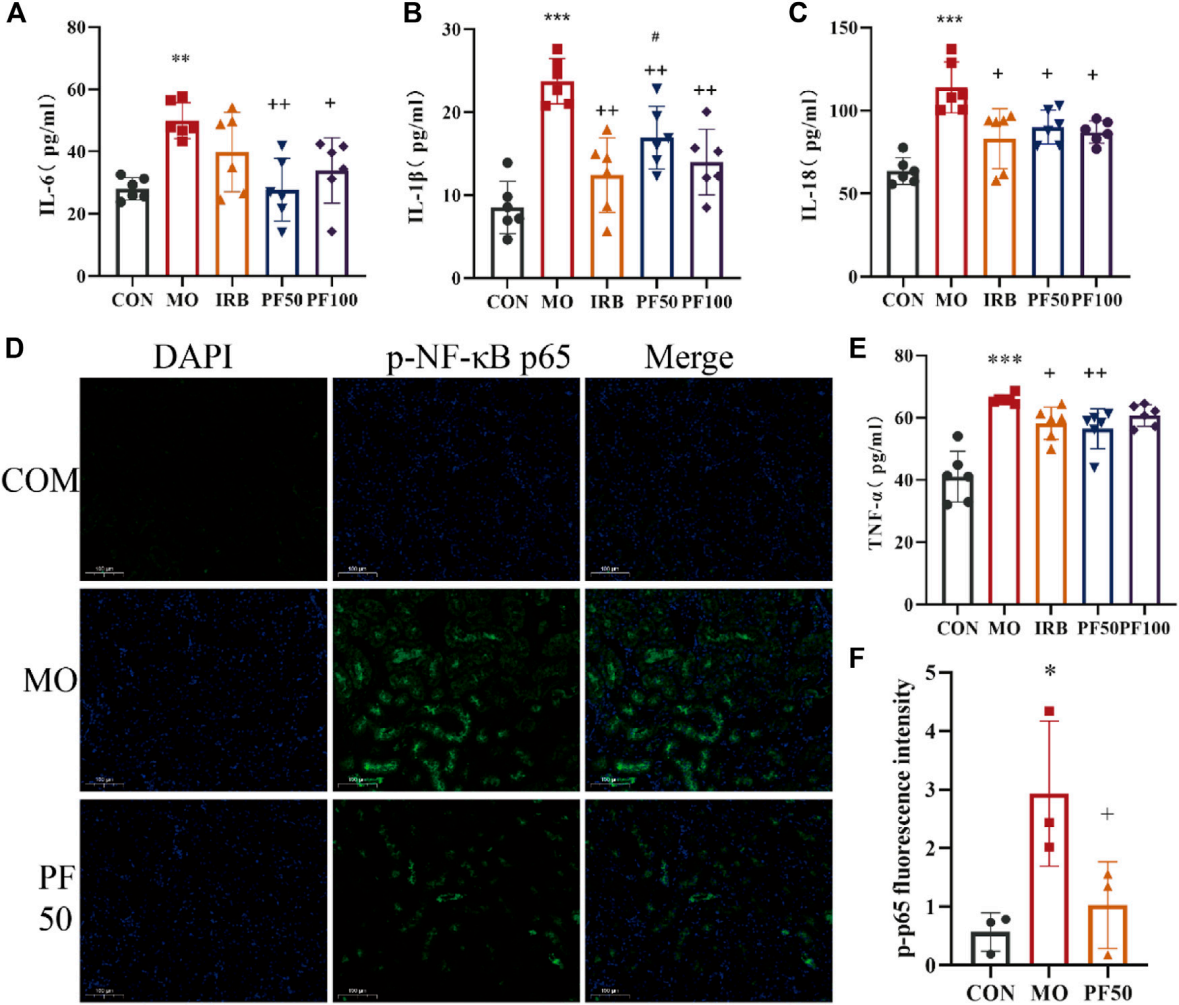
PF attenuates the inflammatory response in DN rats. PF effects on serum IL-6 **(A)**, IL-18 **(B)**, TNF-α **(C)**, and IL-1β **(D)** in DN rats (n = 6). Immunofluorescence assay for p-p65 protein expression (Calculate the average fluorescence intensity using ImageJ) **(E, F)** (n = 3). Compared with the control group, ^#^ *p* < 0.05, ^##^ *p* < 0.01, ^###^ *p* < 0.001; compared with the model group, ^*^ *p* < 0.05, ^**^ *p* < 0.01, ^***^ *p* < 0.001. Data are expressed as ‾x±SD, *p < 0.05, **p < 0.01, ***p < 0.001 relative to the control group; ^+^p< 0.05, ^++^ *p*< 0.01, ^+++^ *p*< 0.001 compared to the model group; #*p* < 0.05, compared to the IRB group.

To investigate the role of inflammation in the protective effects of PF against hyperglycemia-induced kidney damage, we measured pro-inflammatory cytokine levels in serum using an ELISA kit. The results showed that the serum levels of TNF-α, IL-6, IL-1β, and IL-18 were elevated in the model group rats *versus* control rats. In contrast, PF decreased the expression levels of TNF-α, IL-6, IL-1β, and IL-18 (Figure 5).

### 3.5 PF modulates the AGEs/RAGE/NF-κB/NLRP3 pathway to attenuate inflammatory responses in DN rats

**FIGURE 6:**
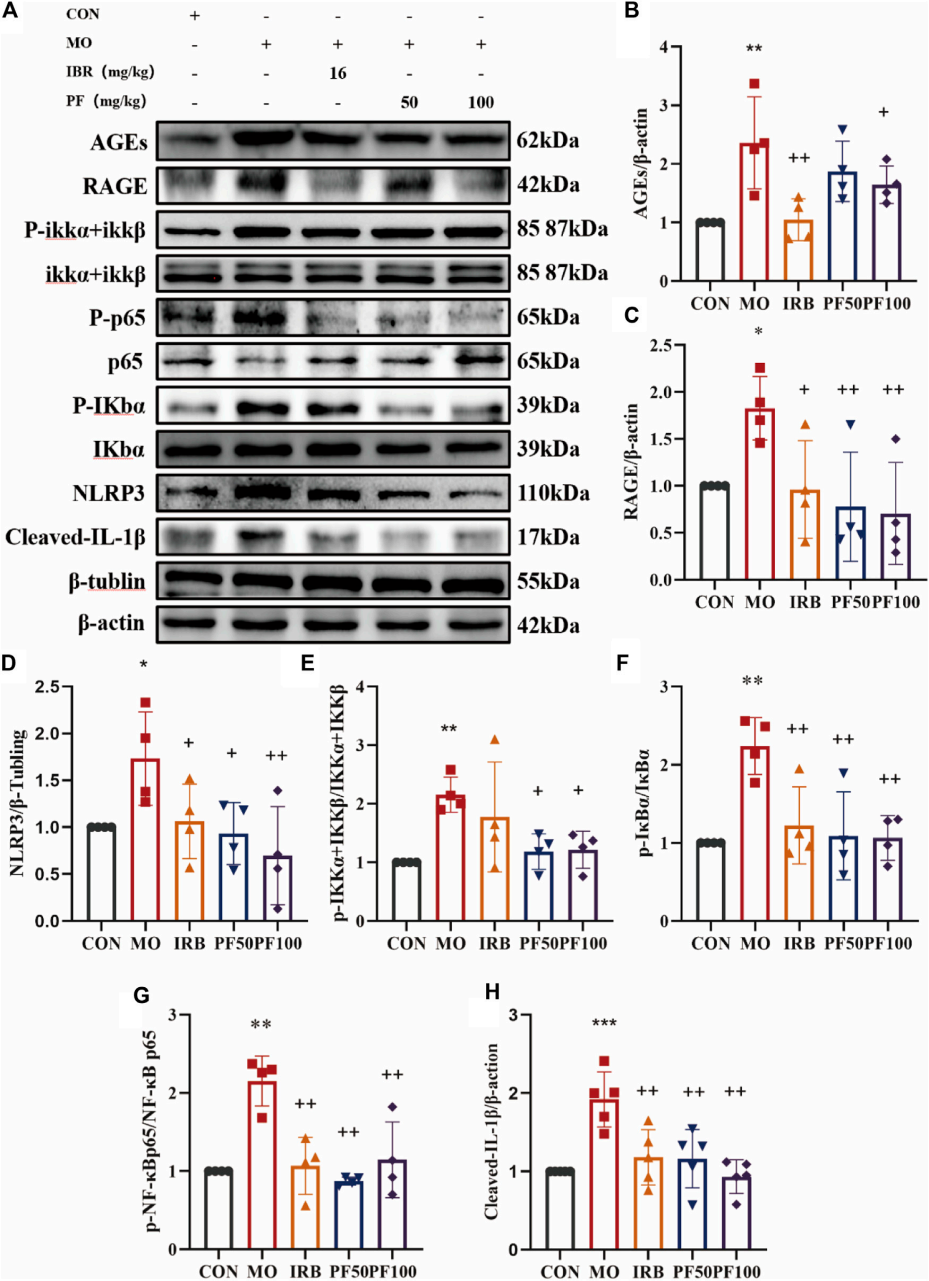
PF modulates AGEs/RAGE/NF-κ B/NLRP3 pathway to reduce inflammation in DN rats **(A)**. Western blotting was performed to determine the AGEs **(B)**, RAGE **(C)**, NLRP3 **(D)**, p-IKK α + IKK β/IKKα + IKKβ **(E)**, p-IκB α/IκBα **(F)**, p-NF-κB p65/NF-κB p65 **(G)**, Cleaved IL- Iβ **(H)** protein expression levels (n = 4). Data are expressed as ‾x±SD, *p < 0.05, **p < 0.01, ***p < 0.001 relative to the control group; ^+^ *p* < 0.05, ^++^ *p* < 0.01, ^+++^ *p* < 0.001 compared to the model group.

Based on the KEGG pathway analysis performed in the preliminary stage of this study, the AGEs/RAGE/NF-κB/NLRP3 pathway, which is related to inflammation and DN, was selected for experimental validation. As shown in Figure 6, AGEs were increased in the kidneys of rats in the model group after high glucose injury, and the activation of RAGE receptors led to increased protein levels of p-IKKα and p-IKKβ. Accordingly, IκB, a downstream target of activated IKK proteins, was phosphorylated, and p-NF-κB-p65, another downstream target, showed increased expression. Meanwhile, activation of the NF-κB signaling activation led to downstream activation of the NLRP3 inflammsome and elevated IL-1 β protein expression (Figure 6).

### 3.6 PF reverses high glucose-induced injury and inflammation in rat podocytes

**FIGURE 7:**
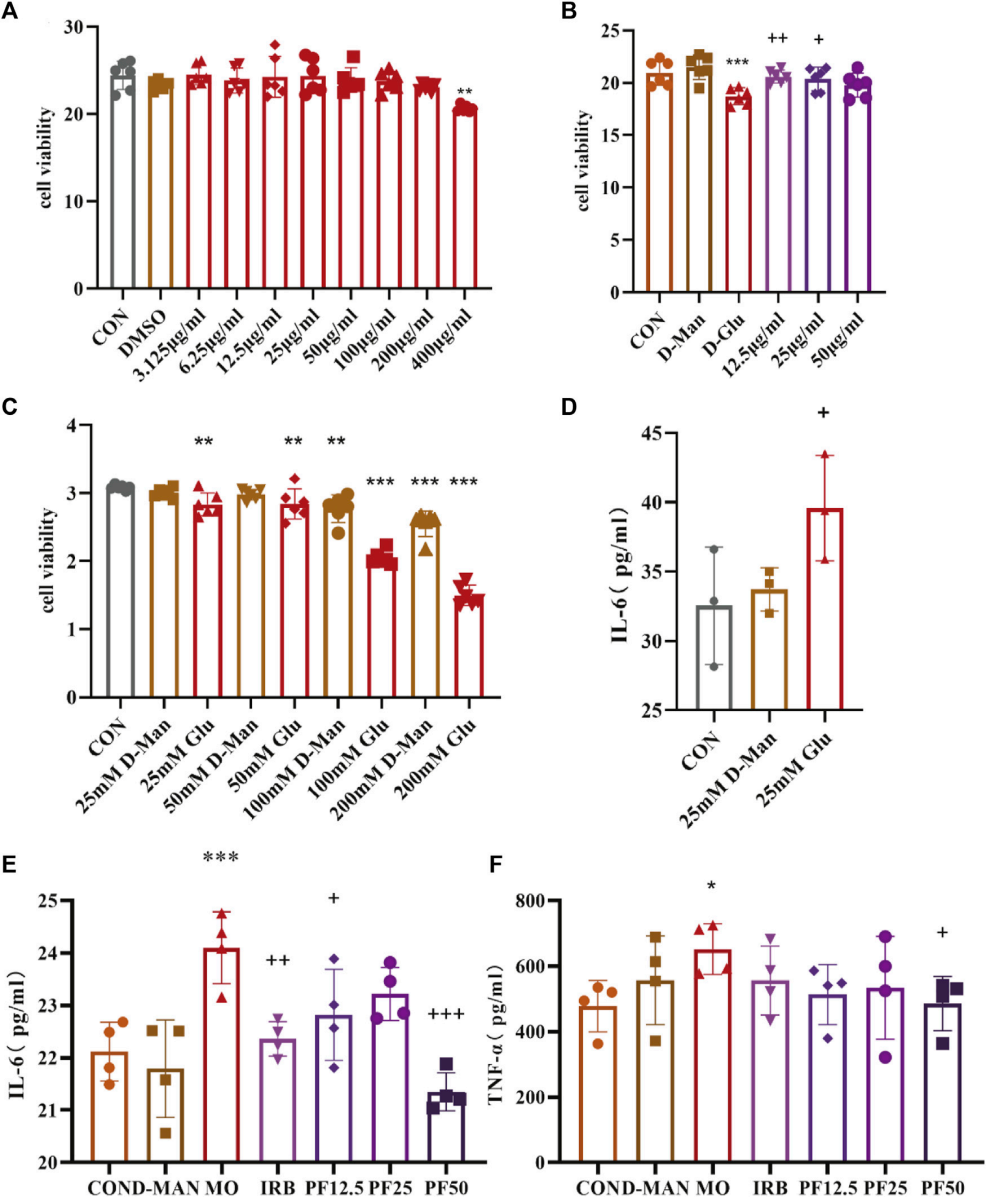
PF attenuates the inflammatory response of podocyte in rats with high glucose injury. Effects of different concentrations of PF on podocyte **(A)**. The effect of PF on high glucose-injured podocytes **(B)**. Degree of podocyte injury by different concentrations of glucose **(C)**.25 mmol/L glucose caused inflammatory response in podocytes **(D)**. Effect of PF on cellular inflammatory factors TNF-α, IL-6 in podocyte supernatant **(E, F)**. Data are expressed as ‾x±SD, *p < 0.05, **p < 0.01, ***p < 0.001 relative to the control group; ^+^ *p*< 0.05, ^++^ *p*< 0.01, ^+++^ *p*< 0.001 compared to the model group.

CCK8 assay results showed that podocyte viability decreased significantly decreased after 24 h of incubation in 25 mM glucose environment. However, PF improved the viability of high glucose-induced rat renal podocytes at concentrations of 12.5 μg/mL, 25 μg/mL, and 50 μg/mL. To assess inflammation, concentrations of secreted pro-inflammatory cytokines were measured in cell supernatants by ELISA. 25 mM glucose significantly increased levels of IL-6 and TNF-α in cell supernatants. However, these changes were reversed by PF treatment (Figure 7).

**FIGURE 8:**
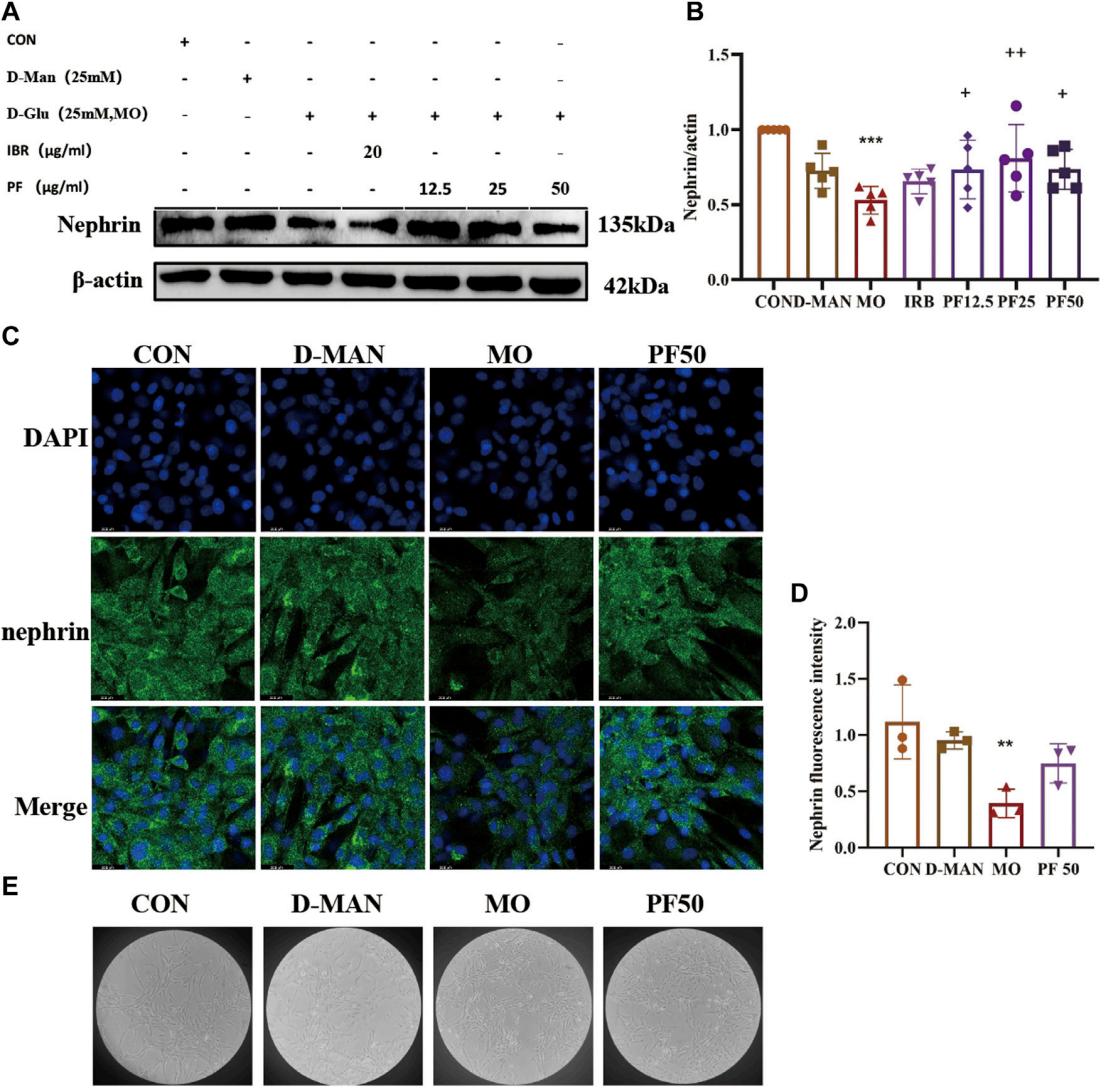
Effect of high glucose on podocytes. The expression level of nephrin was determined by Western blotting **(A, B)** (n = 4). The expression of nephrin was detected by immunofluorescence **(C, D)** (n = 3). cellular morphology **(E)**. Data are expressed as ‾x±SD, *p < 0.05, **p < 0.01, ***p < 0.001 relative to the control group; ^+^ *p*< 0.05, ^++^ *p*< 0.01, ^+++^ *p*< 0.001 compared to the model group.

To investigate the integrity of cell-cell junctions, the expression of nephrin in rat kidney podocytes was examined by immunofluorescence. Cellular nephrin expression was reduced after culture in 25 mM glucose, an effect that was attenuated by PF (Figure 8).

### 3.7 PF inhibits the cellular inflammatory response in rat renal podocytes by modulating the NF-κ B/NLRP3 signaling pathway

**FIGURE 9:**
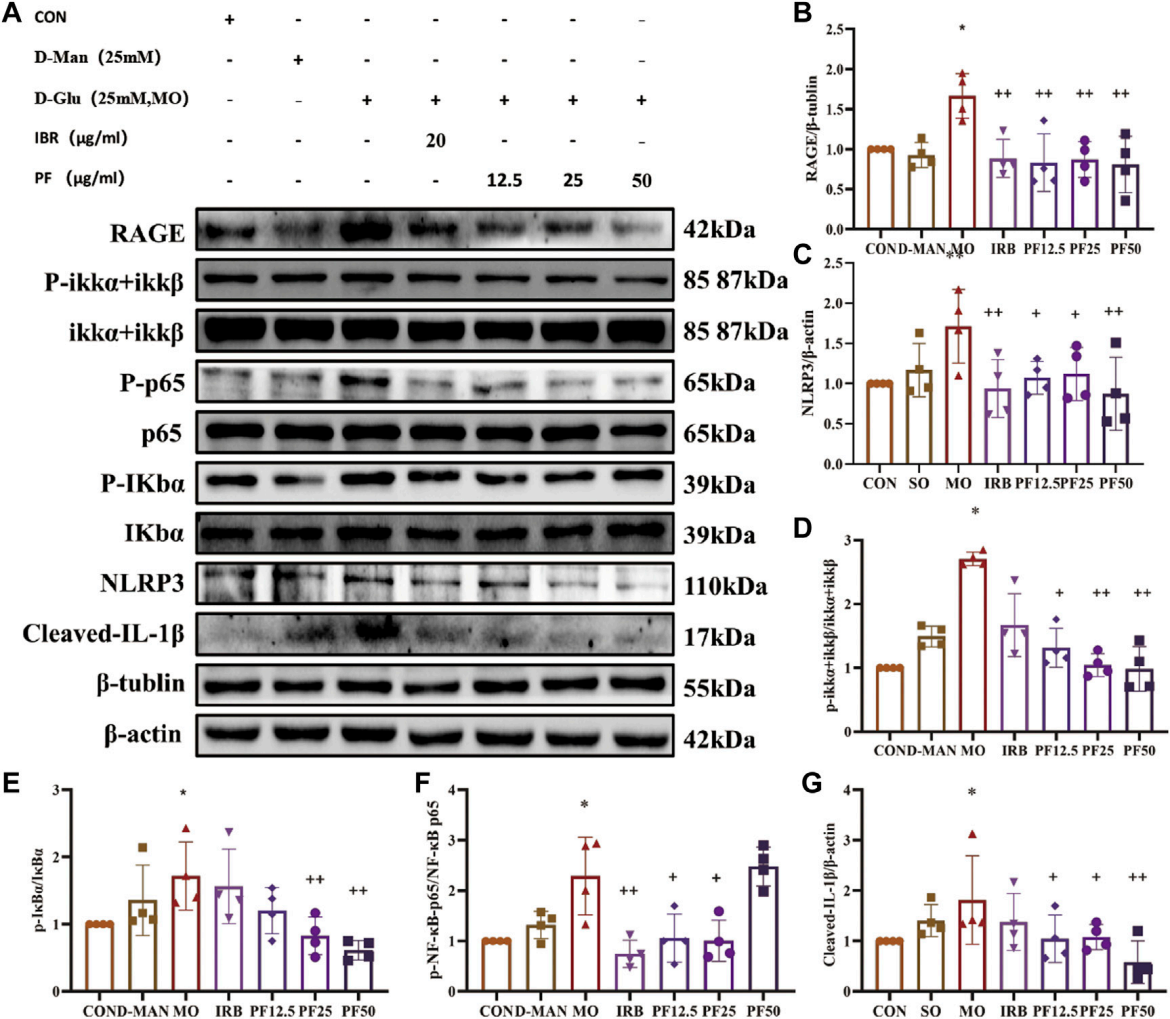
PF regulates AGEs/RAGE/NF-κ B/NLRP3 pathway to attenuate the inflammatory response in podocytes **(A)**. Western blotting was performed to determine the RAGE **(B)**, NLRP3 **(C)**, p-IKK α + IKK β/IKKα + IKKβ **(D)**, p-IκB α/IκBα **(E)**, p-NF-κB p65/NF-κB p65 **(F)**, Cleaved IL- Iβ **(G)** protein expression levels (n = 4). Data are expressed as ‾x±SD, *p < 0.05, **p < 0.01, ***p < 0.001 relative to the control group; ^+^ *p* < 0.05, ^++^ *p* < 0.01, ^+++^ *p* < 0.001 compared to the model group.

To investigate the mechanism by which PF inhibited the inflammatory response of rat renal podocytes, Western blotting was performed to measure the expression of key intracellular molecules involved in inflammatory signaling (p-IKKα + IKKβ, p-IκBα, p-NF-κB-p65, NLRP3, and cleaved-IL-1β). As shown in Figure 9, PF reversed the high glucose-induced increase in p-IKKα + IKKβ, p-NF-κB-p65, and p-IκBα expression, while decreasing NLRP3 and cleaved-IL-1β expression. These results suggest that PF can attenuate cellular inflammation under high glucose conditions by inhibiting the RAGE/NF-κB pathway (Figure 9).

**FIGURE 10:**
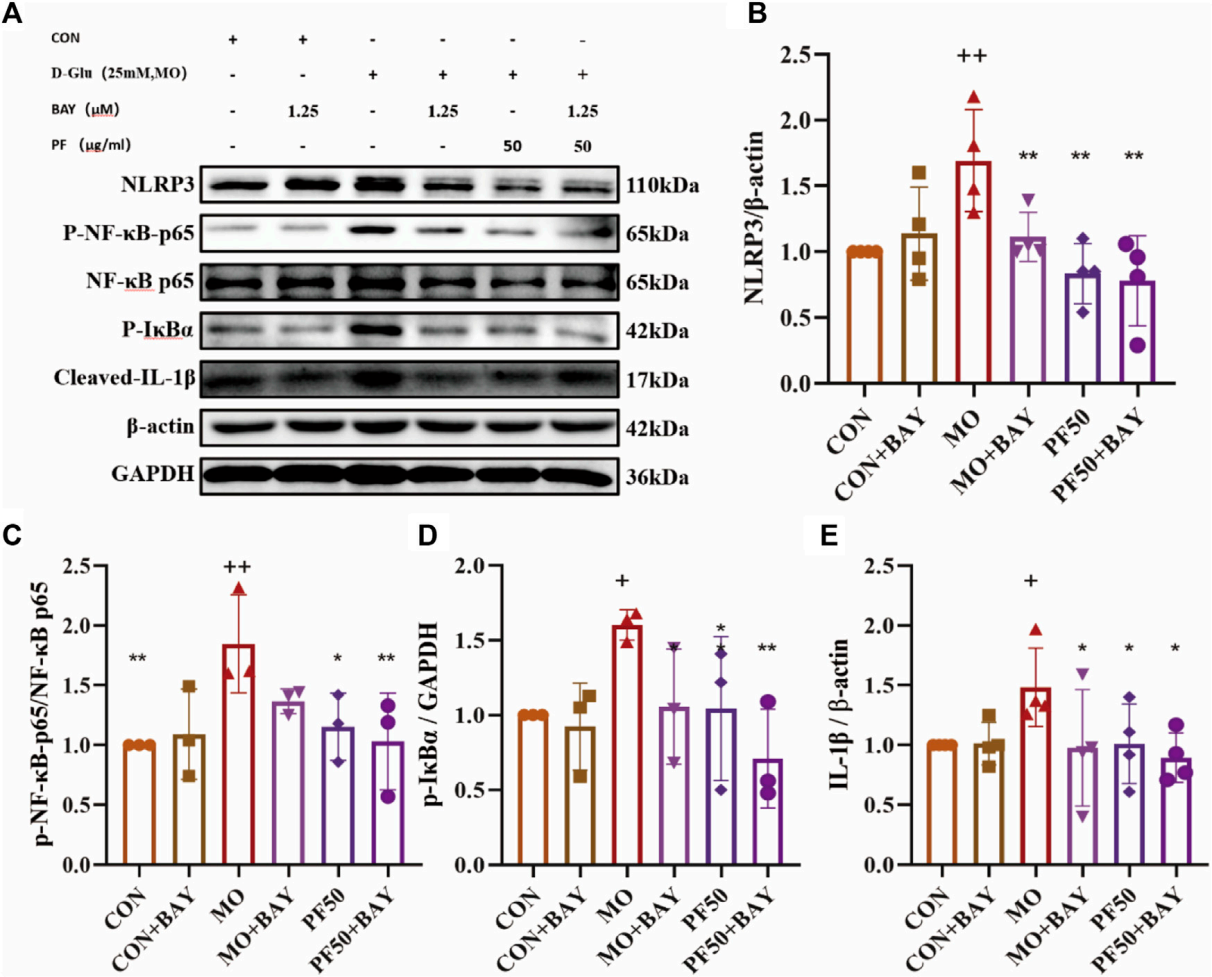
PF regulates the NF-κB/NLRP3 signaling pathway to attenuate the inflammatory response of podocytes **(A)**. The levels of NLRP3 **(B)**, p-NF-κB p65/NF-κB p65 **(C)**, p-IκBα **(D)**, and Cleaved IL- Iβ **(E)** protein expression were determined by Western blotting (n = 4,3). Data are expressed as ‾x±SD, *p < 0.05, **p < 0.01, ***p < 0.001 relative to the control group; ^+^ *p* < 0.05, ^++^ *p* < 0.01, ^+++^ *p* < 0.001 compared to the model group.

To further explore the mechanism of PF’s protective effect of PF on podocytes, cells were additionally treated with the NF-κB inhibitor BAY 11–7821. In high glucose-treated podocytes, PF and BAY 11–7821 inhibited the expression of p-NF-κB p65, p-IκBα, NLRP3, and Cleaved-IL-1β protein expression (Figure 10). In summary, PF attenuates podocyte injury caused by high glucose by inhibiting AGE/RAGE-mediated inflammation.

## 4 Discussion

DN is one of the leading causes of end-stage renal disease worldwide. Currently, treatment of DN focuses on controlling chronic hyperglycemia and hypertension in order to ameliorate renal injury (Samsu, 2021), but these traditional approaches to treating DN do not always achieve satisfactory results. Simply controlling blood glucose is not enough to relieve DN (Tang et al., 2021), and the use of ACEIs or ARBs in some patients with normal blood pressure does not completely stop the progression of nephropathy (de Boer et al., 2007; Xiang et al., 2016). Therefore, there remains an important need to explore the mechanisms of DN pathogenesis and develop novel therapies.

The pathophysiology of DN involves a variety of molecular mechanisms, including oxidative stress, inflammation, and dysregulation of the renin-angiotensin system (Yaribeygi et al., 2019). Recent evidence suggests that the cell surface receptor RAGE plays a central role in the regulation of immune responses and inflammation (Bierhaus and Nawroth, 2009). Under conditions of diabetes, excess glucose increases the formation of AGEs, which interact with the cell surface receptor RAGE, leading to the activation of certain signaling pathways and overproduction of other inflammatory mediators. This cascade of inflammatory signaling ultimately results in podocyte damage, which is a key factor in the pathogenesis of DN (Yu et al., 2018; Amorim et al., 2019; Heyman et al., 2022). Mechanistically, the binding of AGEs to RAGE induces renal inflammation through the sustained activation of the pro-inflammatory transcription factor NF-κB, which causes increased production of pro-inflammatory cytokines, including IL-6, IL-18, IL-1β, and TNF-α. These cytokines trigger signaling events that lead to local and systemic inflammation, glomerular and tubular pathology, and ultimately proteinuria (Amorim et al., 2019; Ding et al., 2019; Yaribeygi et al., 2019).

The presence of proteinuria in DN is indicator of damage to the glomerular filtration barrier, causing protein loss and, thus, proteinuria. Podocytes are terminally differentiated polymorphic cells that attach to the surface of the GBM, and are characterized by their distinctive foot processes. The cell-cell junctions between the foot processes, called the slit diaphragms (SDs), are an important structure that helps to connect neighboring foot processes and forms a filtration barrier involved in the maintenance of normal renal function (Garg, 2018). Nephrin and podocin on the SD have important roles in maintaining normal glomerular filtration function, and are key markers of podocyte injury. Nephrin is a transmembrane protein, while podocin is an actin cytoskeleton protein; both are responsible for cell-cell connectivity among podocytes and play key roles in the development of renal injury (Verma et al., 2018; Zeng and Szeto, 2021). Notably, podocytes are highly susceptible to damage as they are highly differentiated with limited renewal capacity. As podocytes represent the main compoments of the glomerular filtration barrier, any pathogenic alteration in their cell structure and protein expression can result in compromised renal function (Tao-Tao and Min-Li, 2013; Audzeyenka et al., 2021).

A variety of approaches have been proposed for the treatment of DN, including *in vivo* and *in vitro* studies into the potential of several active ingredients for relieving DN. PF, as a compound chemically synthesized based on the structure and activity of ferulic acid and chuanxiongxizine, is an ideal drug candidate because it is a single ingredient, has predictable drug-drug interactions, demonstrates a favorable safety profile, and may be produced at low cost (Li et al., 2021). The current clinical use of PF has been shown to significantly reduce the levels of renal injury markers such as Scr and BUN when used alone or in combination, which is of great clinical significance (Han et al., 2023). However, there is a scarcity of studies reporting the use of PF to treat DN, and most existing studies co-administered PF with other drugs such as ACEIs/ARB; hence, the efficacy of PF alone and its mechanism of action have not yet been explored. Irbesartan belongs to the ARB class of antihypertensive drugs, and when used in the treatment of diabetic nephropathy, it can reduce the glomerular filtration pressure and play a role in protecting the kidneys (Zhao Qing et al., 2024). Irbesartan was used as a positive control in the text of this study to provide a reliable control standard for the efficacy evaluation of ferulic acid piperazine in the treatment of diabetic nephropathy. In current study, preliminary network pharmacological analysis demonstrated that PF may act through the AGE-RAGE signaling pathway, suggesting that RAGE activation may promote the development of inflammation in DN. Given that the clinical application of PF can reduce renal injury, inhibit inflammation (Lei et al., 2022), and reduce cytokine levels (Li et al., 2022), this study aimed to investigate whether PF inhibits the inflammatory response through AGE-RAGE signaling, thereby alleviating DN.

AGEs in DN patients commonly accumulate in renal cells, of which podocytes are specifically affected. AGEs exacerbate renal cell damage by binding to and activating the RAGE receptor, which promotes intracellular signaling, such as the NF-κB pathway (Huang et al., 2015). NF-κB has been shown to regulate the expression of pro-inflammatory cytokines (Cortez et al., 2013), and is activated in podocytes in response to an inflammatory microenvironment (Bao et al., 2015). Under unstimulated conditions, NF-κB interacts IκB, which inhibits NF-κB activity. However, in a microenvironment of inflammation and oxidative damage, IKK proteins are phosphorylated and activated, resulting in the phosphorylation and degradation of IκB and the phosphorylation and activation of NF-κB (Dong et al., 2022). NF-κB activation in turn activates the NLRP3 inflammasome and promotes the maturation of its downstream inflammatory cytokines IL-1β and IL-18 (Ganz et al., 2014; Hassanein et al., 2021), resulting in inflammatory damage.

In the present study, PF significantly ameliorated renal histopathological changes triggered by STZ-induced diabetes after 12 weeks of continuous administration, while decreasing inflammatory markers in the kidneys, reducing urinary protein output, and preserving functional glomerular filtration in DN rats. Both PF50 mg/kg and PF100 mg/kg could better alleviate the renal injury in rats with diabetic nephropathy. And like Irbesartan, they showed better efficacy in BUN, cys-c, and inflammatory factors. Overall, the PF50 mg/kg group could more obviously reduce KIM-1, decrease the glycogen expression area, and improve the renal pathological injury, showing a better renal protective effect. *In vitro*, PF attenuated the inflammatory response in high glucose-challenged podocytes, and restored podocyte nephrin and podocin expression. Mechanistically, PF inhibited NF-κB inflammatory pathway activation by inhibiting upstream AGE-RAGE signaling. Hence, the regulation of the AGE/RAGE signaling pathway and its effects on the NF-κB/NLRP3 inflammatory pathway at least partly explain the phenotypic improvements in the DN rat kidneys and high glucose-treated rat podocytes. This study provides new insights into the renoprotective effects of PF and its mechanism of action in DN, guiding the selection and development of novel drug therapies for clinical DN treatment.

## 5 Conclusion

**FIGURE 11:**
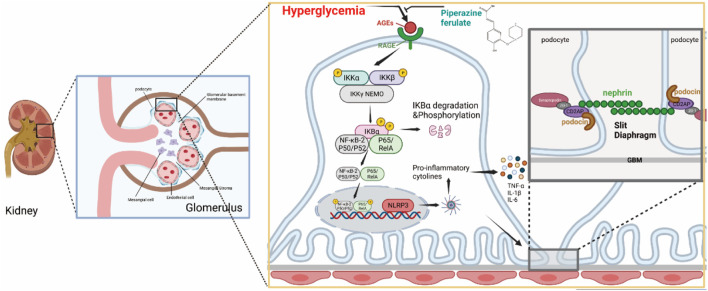
PF regulates AGEs/RAGE/NF-κ B/NLRP3 pathway to attenuate the inflammatory response in podocytes.

In conclusion, our study suggests that PF can inhibit the inflammatory response and play a protective role against renal injury in DN. This may be related to the fact that PF reduces the formation of AGEs and inhibits RAGE receptor activation, thereby suppressing the activation of NF-κB as well as the downstream NLRP3 inflammasome. Therefore, we propose that PF is a potential candidate for the treatment of DN due to its ability to protect the kidney against high glucose-related damage by suppressing inflammation and preserving podocytes (Figure 11).

## Data Availability

The original contributions presented in the study are included in the article/Supplementary Material, further inquiries can be directed to the corresponding authors.

## Abbreviations

AGEs, Advanced glycation end products; BC, Betweenness Centrality; BUN, Blood Urea Nitrogen; CC, Closeness Centrality; CCR, Endogenous Creatinine Clearance; cys-c, Cystatin C; DC, Degree Centrality; DM, Diabetes Mellitus; DN, Diabetic Nephropathy; EC, Eigenvector Centrality; FBG, Fasting Blood Glucose; GBM, Glomerular Basement Membrane; GFR, Glomerular Filtration Rate; IOD, Integrated Optical Density; IL-1β, Interleukin-1β; IL-6, Interleukin-6; IL-18, Interleukin-18; KIM-1, Kidney Injury Molecule-1; MMP-9, Matrix metalloproteinase 9; NLRP3, NOD-like Receptor Protein 3; NF-κB, Nuclear Factor-κB; PF, Piperazine Ferulate; T1DM, Type 1 diabetes mellitus.
